# A Systematic Review of Human and Robot Personality in Health Care Human-Robot Interaction

**DOI:** 10.3389/frobt.2021.748246

**Published:** 2021-09-17

**Authors:** Connor Esterwood, Lionel P. Robert

**Affiliations:** ^1^ School of Information, University of Michigan, Ann Arbor, MI, United States; ^2^ Robotics Institute, University of Michigan, Ann Arbor, MI, United States

**Keywords:** personality, healthcare, HRI (human robot interaction), health care HRI, healthcare robots, big five personality, systematic review

## Abstract

Robots have become vital to the delivery of health care and their personalities are often important to understanding their effectiveness as health care providers. Despite this, there is a lack of a systematic overarching understanding of personality in health care human-robot interaction. This makes it difficult to understand what we know and do not know about the impact of personality in health care human-robot interaction (H-HRI). As a result, our understanding of personality in H-HRI has not kept pace with the deployment of robots in various health care environments. To address this, the authors conducted a literature review that identified 18 studies on personality in H-HRI. This paper expands, refines, and further explicates the systematic review done in a conference proceedings [see: Esterwood (Proceedings of the 8th International Conference on Human-Agent Interaction, 2020, 87–95)]. Review results: 1) highlight major thematic research areas, 2) derive and present major conclusions from the literature, 3) identify gaps in the literature, and 4) offer guidance for future H-HRI researchers. Overall, this paper represents a reflection on the existing literature and provides an important starting point for future research on personality in H-HRI.

## 1 Introduction

Robots are one solution to the growing shortage of health care workers that the recent coronavirus disease 2019 (COVID-19) outbreak has only exacerbated. Even before the COVID-19 pandemic, the demand for health care services was expected to far outpace the availability of health care workers (Broadbent et al., 2009; Robinson et al., 2014; Deutsch et al., 2019). This shortage is largely attributable to the projected increase in health care workers older than 60 years, which is expected to rise from 12.3 to 22.0% of the global population by 2050 (WHO, 2016; Bhandari, 2020). Robots as health care workers is one solution to addressing the shortage of health care workers (Broadbent et al., 2009; Bogue, 2011; Robinson et al., 2014; Deutsch et al., 2019). During this pandemic, robots have been utilized to conduct health screenings, transport medical goods, and even direct patient care (Kimmig et al., 2020; Murphy et al., 2020; Tan and Seetharaman, 2020; Yang et al., 2020; Zeng et al., 2020; Esterwood and Robert, 2021). This trend is only set to grow as the global medical robotic market, valued in 2018 at $2,257.8 million is projected to grow annually at the rate of 21.5% and reach $10,710.6 million by 2026 (Fortune Business Insights, 2019). Given the increased and projected deployment of robots as health care workers, it is clear that this is an important area of study.

Personality—both the patient’s and the robot’s—has been identified as a key predictor of whether a patient will accept a robotic health care worker (Esterwood and Robert, 2020). This research is emerging across such diverse and distinct fields of study as human-computer interaction (HCI), human-robot interaction (HRI), human factors in engineering (HFE) and cognitive and social psychology. Unfortunately, the literature is fragmented. This makes it difficult to understand what we know and to identify what we do not know about personality and health care robots. It also creates a barrier to the organization and integration of potential design solutions. At present, it is difficult to know, for example, whether there is a growing consensus regarding which personality traits a robot should or should not have as a health care worker. The result is a fragmented and incoherent view of both the research area and its related design space. This necessitates a need to reflect on what has been done in this area and to contemplate what still needs to be done.

To accomplish this, our review offers three contributions to the literature. First, similar to Esterwood and Robert (2020) this paper presents the results of a systematic literature review on personality in H-HRI. The results of this systematic review organize and highlight the findings across the literature on the topic of personality in H-HRI. However, unlike Esterwood and Robert (2020) this paper also compares the findings to the broader personality health care literature. Second, this paper highlights and discusses the various methodologies, outcomes, and samples that have been employed. In doing so, the review goes beyond Esterwood and Robert (2020), with regards to depicting the choices taken by scholars studying personality in H-HRI. Finally, this review identifies several important understudied areas vital to advancing our understanding over and above those identified by Esterwood and Robert (2020). Knowledge of these gaps can help inform and guide researchers in the burgeoning field of H-HRI.

## 2 Background

### 2.1 Trait-Based Approach to Personality Psychology

Personality can be defined as an individual’s “characteristic pattern of behaviour in the broad sense (including thoughts, feelings, and motivation)” (Baumert et al., 2017, p. 527). There are several schools of thought regarding human personality (see McMartin, 2016, for a review). One of the most popular, the trait-based perspective, views traits as the primary mechanism by which personality manifests. A trait can be considered “a component or distinguishing characteristic of an individual’s personality that is stable across time and external situations” (Ellis et al., 2008, p. 219). Personality traits are factors that can predict an individual’s attitudes and by extension behavior (Robert, 2018). Although there are numerous sets of traits, the most used in the HRI community are the Big Five personality traits (Robert, 2018; Robert et al., 2020).

The Big Five personality traits consist of extroversion, agreeableness, conscientiousness, neuroticism, and openness to experience. Extroversion is the degree to which an individual is outgoing, assertive, talkative, and sociable (Rhee et al., 2013). The opposite of extroversion is introversion which is the degree to which a person is quiet, reserved, or shy. Agreeableness can be defined as the extent to which someone is cooperative and friendly (Peeters et al., 2006). The opposite of an agreeable individual is an uncooperative exemplified by cold, callous, selfish, hostile, and competitive characteristics.

Conscientiousness is the extent that a person is careful, deliberate, and self-aware of their actions (Tasa et al., 2011). The opposite of a conscientious person is an unreliable, careless, impulsive, and disorganized person. Neuroticism is the degree to which someone is easily angered, not well-adjusted, insecure, or lacking in self-confidence (Driskell et al., 2006). The opposite of a neurotic individual is an individual who is emotionally stable. These individuals elicit calm behaviors, lower degrees of emotionality, and generally high ability to cope with stress. Finally, a person who is open to experience is highly imaginative, curious, and broadminded (McCrae and Costa Jr, 1997). The opposite of a person with openness to experience is someone who is rigid, conservative and conventional in their decision-making processes. A summary of these traits with associated synonymous and antonymous descriptive characteristics based on the work of (John and Srivastava, 1999) is available in Table 1.

**TABLE 1:** Table displaying synonyms and antonyms for the Big Five personality traits based on John and Srivastava (1999).

Trait	Synonymous with	Antonymous with
Extroversion vs. Introversion	Sociable, assertive, enthusiastic, energetic, forceful, talkative	Introversion: Quiet, reserved, shy, retiring
Agreeableness vs. Uncooperative	Warm, modest, kind, appreciative, trusting, affectionate, helpful	Uncooperative: Cold, quarrelsome, unfriendly
Conscientiousness (reliable vs. unreliable)	Efficient, organized, thorough, planful, reliable	Unreliable: Careless, irresponsible, frivolous
Neuroticism vs. Stable	Tense, irritable, shy, moody, nervous, high-strung	Stable: Calm, contented, unemotional
Openness to experience vs. Conventionality	Imaginative, intelligent, original, insightful, curious, sophisticated	Conventional: Narrow interests, simple, shallow

The Big Five personality traits have been used to predict employees’ attitudes and behaviors. For example, researchers have examined the use of the Big Five personality traits in the prediction of attitudes and behaviors of employees and customers (Funder, 1997; Fleeson, 2001; Matthews et al., 2003; Saucier, 2008; Boyle et al., 2014). In the health care domain, personality traits like the Big Five have also been examined with a focus on patient and health care provider outcomes.

### 2.2 Personality and Health Care

The research examining the impacts of personality in health care can be organized into two broad themes. Theme one examines the impact of a patient’s personality on health outcomes and experiences. Overall, the results of these studies have found that agreeableness, conscientiousness, and extroversion are positively related to patient outcomes and experiences while neuroticism and openness to experience are negatively related to patient outcomes and experiences (Wikehult et al., 2005; Block et al., 2007; Orom et al., 2009; Kim et al., 2013; Koh et al., 2014; Victorson et al., 2016). Theme two examines the impact of the health care provider’s personality on health outcomes and experiences. Generally, results have found that agreeableness, conscientiousness, extroversion, and openness to experience are positively related to outcomes while neuroticism is negatively related to them (Mohler et al., 2010; Cox, 2012; Fiabane et al., 2013; Ellershaw et al., 2016; Yeh et al., 2016; Taherinejad et al., 2017).

#### 2.2.1 Theme 1: Patient Personality

Patient personality has been shown to be significantly associated with how patients respond to treatment and their overall health care experiences. This line of research relies heavily on patients’ post-treatment quality of life (QoL) as a measure of patient health care outcomes. (Kim et al., 2013; Victorson et al., 2016). QoL is a multidimensional measurement of patient well-being, typically examined in two ways: 1) as one or more of four separate sub-dimensions or 2) as a singular overall measure (Victorson et al., 2016). The four QoL sub-dimensions are: psychological, social relationships, environmental, and physical health and are designed to measure different aspects of life and living (Kim et al., 2013). Post-treatment depression and somatization are other measurements of patients’ responses to treatments (Koh et al., 2014).

Across the literature, there is strong support for some personality traits impacting patient outcomes and not others. For studies that indicated a positive and significant effect, three of the Big Five personality traits have been linked to increased satisfaction, treatment responses, and/or quality-of-life outcomes. First, extroversion has been shown to be related to improvements in the psychological domain of QoL and how patients respond to treatment (Kim et al., 2013; Koh et al., 2014). For example, Koh et al. (2014) found that extroverts tend to have fewer chronic prostatitis symptoms when compared to introverts. Second, agreeableness was positively related to overall QoL (Victorson et al., 2016) as well as three of the four QoL sub-domains (psychological, physical health and social relationships; Kim et al., 2013). Agreeableness was also associated with better treatment responses in terms of lower levels of depression and somatization for patients who received treatment for chronic prostatitis (Koh et al., 2014). Third, conscientiousness was positively related to improvements in the psychological domain of QoL and satisfaction with health care experiences (Block et al., 2007; Kim et al., 2013). Beyond these Big Five personality traits, three additional traits were found to have significant and positive effects on patients’ health care satisfaction. Specifically, social desirability, detachment (Wikehult et al., 2005), and optimism (Orom et al., 2009) were linked to higher levels of satisfaction with the health care experience.

In addition to the positive relationships, two of the Big Five personality traits have been linked to negative relationships. Several studies showed that neuroticism is negatively related to QoL (Kim et al., 2013; Koh et al., 2014; Victorson et al., 2016). For example, Koh et al. (2014) found that for patients who received treatment for chronic prostatitis, neuroticism was associated with increases in the severity of their depression and somatization. Additionally, Kim et al. (2013) examined the QoL of patients recovering from a stroke over a 3 month period and found that the more neurotic patients were, the lower overall QoL levels they exhibited during the recovery period. For openness to experience, patients with higher degrees of openness to experience were less satisfied with their health care experiences (Block et al., 2007). Aside from the Big Five traits, the traits of stress susceptibility and pessimism were also investigated and were found to be linked to lower satisfaction (Wikehult et al., 2005; Orom et al., 2009). A summary of patient personality traits and their impact on health-care-related outcomes is presented in Table 2.

**TABLE 2:** Summary of patient’s personality traits on health-care-related outcomes.

Patient personality			
Study	Personality trait	Finding	Outcome
Kim et al. (2013)	Extroversion (BFI)	Sig positive	Higher quality of life
Koh et al. (2014)			
			Better treatment response
Kim et al. (2013)	Agreeableness (BFI)	Sig positive	Higher quality of life
Victorson et al. (2016)			
Koh et al. (2014)			Better treatment response
Kim et al. (2013)	Conscientiousness (BFI)	Sig positive	Higher quality of life
Block et al. (2007)			Higher satisfaction
Kim et al. (2013)	Neuroticism (BFI)	Sig negative	Lower quality of life
Victorson et al. (2016)			
Koh et al. (2014)			Worse treatment response
Block et al. (2007)	Openness (BFI)	Sig Negative	Lower Satisfaction
Wikehult et al. (2005)	Social Desirability	Sig Positive	Higher Satisfaction
Wikehult et al. (2005)	Detachment	Sig Positive	Higher Satisfaction
Orom et al. (2009)	Optimism	Sig Positive	Higher Satisfaction
Wikehult et al. (2005)	Stress Susceptibility	Sig Negative	Lower Satisfaction
Orom et al. (2009)	Pessimism	Sig Negative	Lower Satisfaction

#### 2.2.2 Theme 2: Health Care Provider Personality

Personality has shown to be a strong predictor of health care employee performance. In the literature, performance has been considered in terms of worker efficiency (Yeh et al., 2016), a composite of multiple constructs (adaptivity, pro-activity, and proficiency; Ellershaw et al., 2016), an extension of workplace engagement (Molero Jurado et al., 2020) and the result of greater organizational commitment (Taherinejad et al., 2017). Studies of this kind have primarily focused on the Big Five personality traits and have primarily sampled nurses from a range of health care environments.

Several provider personality traits have been identified as having significant and positive effects. Agreeableness has a positive association with performance. For example, Molero Jurado et al. (2020) found that the more agreeable nurses were, the more workplace engagement they exhibited which led to increased performance. Similarly, extroversion has been positively associated with performance (Ellershaw et al., 2016; Taherinejad et al., 2017; Molero Jurado et al., 2020). In particular, Taherinejad et al. (2017) found that extroverted health care providers exhibited more organizational commitment, which also led to higher performance. Furthermore, four studies found that conscientiousness had a positive relationship with performance (Ellershaw et al., 2016; Yeh et al., 2016; Taherinejad et al., 2017; Molero Jurado et al., 2020). For example, Ellershaw et al. (2016) examined performance via individual: proficiency, adaptivity, and pro-activity. Results showed a strong positive link between these performance measures and conscientiousness. Finally, two studies examined openness to experience. These studies identified a positive relationship between health care providers’ openness to experience and their performance (Ellershaw et al., 2016; Molero Jurado et al., 2020). Specifically, nurses who were more open to experience were also more proficient, adaptive, proactive and committed (dedicated) to their organization (Ellershaw et al., 2016; Molero Jurado et al., 2020).

Other non-Big Five personality traits have also been shown to have significant and positive associations with health care provider performance. For example, Cox (2012) found that intuition and thinking, measured via the Myers Briggs Test (MBT), were both positively associated with performance. Fiabane et al. (2013) studied the impacts of “Type A” and “Type B” personalities on workplace engagement in hospital staff (registered nurses, nurse aides, physicians, and physiotherapists). Workplace engagement in this study was defined as a combination of three variables: energy, involvement, and professional efficacy. This study found that staff with “Type A” personalities had more workplace energy than hospital staff with “Type B” personalities.

Neuroticism is the one exception to the positive effects of traits. Neuroticism has shown a significant and negative relationship with performance. Specifically, neuroticism in nurses has been significantly linked to lower performance measured as efficiency (Yeh et al., 2016). Table 3 summarizes these findings from the human-human health care literature. Across both of these themes, it would appear that human personality plays a significant role in health care.

**TABLE 3:** Summary of provider’s personality traits on related outcomes.

Provider personality			
Study	Personality trait	Finding	Outcome
Ellershaw et al. (2016)	Extroversion (BFI)	Sig positive	Higher performance
Molero Jurado et al. (2020)			
Taherinejad et al. (2017)			
Molero Jurado et al. (2020)	Agreeableness (BFI)	Sig positive	Higher performance
Taherinejad et al. (2017)			
Yeh et al. (2016)	Conscientiousness (BFI)	Sig positive	Higher performance
Ellershaw et al. (2016)			
Molero Jurado et al. (2020)			
Taherinejad et al. (2017)			
Yeh et al. (2016)	Neuroticism (BFI)	Sig Negative	Lower Performance
Ellershaw et al. (2016)	Openness (BFI)	Sig positive	Higher performance
Molero Jurado et al. (2020)			
Taherinejad et al. (2017)			
Cox (2012)	Intuition and Thinking	Sig Positive	Higher Performance
Fiabane et al. (2013)	Type A Personality	Sig Positive	Higher Performance

Based on our review, although not comprehensive, three trends emerged. The first is a link between positive outcomes and the traits of agreeableness, conscientiousness, and extroversion for both providers and patients (Block et al., 2007; Kim et al., 2013; Koh et al., 2014; Ellershaw et al., 2016; Victorson et al., 2016; Yeh et al., 2016; Taherinejad et al., 2017; Molero Jurado et al., 2020). The second is the observation that neuroticism negatively impacts outcomes for both patients and providers (Kim et al., 2013; Koh et al., 2014; Victorson et al., 2016; Yeh et al., 2016). Third, openness to experience has been linked to positive outcomes for providers but negative outcomes for patients. Specifically, providers high in openness to experience appear to perform better (Ellershaw et al., 2016; Taherinejad et al., 2017; Molero Jurado et al., 2020), but patients high in openness to experience have lower treatment satisfaction and worse experiences (Block et al., 2007). The prior literature supports the assertion that personality is an important factor in the health care domain. This might explain why HRI researchers and designers have sought to leverage personality in understanding effective H-HRI.

### 2.3 HRI, Personality, and Outcomes

Across the general literature on the subject of HRI, there appears to be a growing consensus that personality impacts users’ interactions with robots. This literature can be divided into three broad categories of outcomes: performance, social/emotional, and acceptance outcomes. First, performance outcomes research has looked at outcomes like to time spent on a specific task, and compliance. For example, Rossi et al. (2018) found a positive link between openness to experience and humans’ performance on a robot assisted task. Second, social/emotional outcomes such as likability, emotions, and engagement were also examined. One example of such a study is that of Häring et al. (2014), who found a significant relationship between participants’ degree of extroversion and their willingness to trust a robot. Finally, acceptance outcomes are outcomes linked directly to acceptance constructs or related measures of acceptance such as trust, intention to buy, and the Universal Theory of Acceptance and Use of Technology’s constructs. An example of a study of this sort is that of Conti et al. (2017) who identified openness to experience and extroversion as significant predictors of humans’ acceptance of robots. Ultimately, within the HRI literature, there appears to be a growing space carved out for discussions of human personality. Health care robots are only one of many applications of robotics, and as a result we utilize the existing HRI literature as a basis for this review sub-setting studies that have specifically focused their efforts in the health care domain. For a comprehensive review of this broader literature Robert et al., 2020.

## 3 Methods

To identify the academic work related to personality in H-HRI, we conducted a literature review. Below, we provide a step-by-step description of the process involved in the literature review.

### 3.1 Search Process

The literature search employed multiple searches via Google Scholar, ACM Digital Library, IEEE Explore, and Scopus.

### 3.2 Search Terms

For this search we used five search terms: “human,” “robot,” “human-robot interaction,” “HRI,” and “personality.” The results of these searches were manually reviewed on a search engine result page (SERP) basis using our initial inclusion criteria. We paged through the SERPs progressively until no single result on the list met the specified criteria. Results prior to the page with no relevant results were extracted for review while subsequent results were not. Each SERP contained 10–25 results (depending on the database) by default. In total, we found 1,819 results across all of our searches before accounting for duplicate entries.

### 3.3 De-Duplication

We performed several steps to remove duplicate articles. First, we exported search results from Google Scholar in .bib format using the “publish or perish” application (Harzing, 2007) and imported them into R for processing. The other databases’ results were exported using their respective built-in tools. We conducted de-duplication using the revtools package (Westgate, 2019). We identified duplicate articles on the basis of title using fuzzy matching and followed up with manual screening. After duplicates were removed, we were left with 1,069 total unique entries.

### 3.4 Eligibility Criteria

To determine whether a paper met the eligibility criteria, we used a three-stage evaluation approach. The first stage involved assessing whether the paper met the initial eligibility criteria. This consisted of a page-by-page review of search results. The second stage involved assessing whether a paper met the second-level eligibility criteria. This consisted of reading and assessing titles and abstracts. Finally, the third stage involved assessing whether a paper met the third level eligibility criteria, and for this stage we investigated abstracts and full texts. The exclusion criteria were used throughout all steps of this review.

Papers were selected for inclusion if they met three specific criteria. First, studies were required to be classified as articles or academic works that excluded patents and popular press articles. Second, studies were required to be written in English. The reason for excluding non-English-language publications relates to the lack of a specialist or translator on our team, making it difficult to review non-English language publications. The third criterion for our initial eligibility was that the titles or abstracts retrieved must have explicitly mentioned both the term “robot” and “personality.” At the secondary level, papers were selected on the basis of four additional eligibility criteria. First, studies were required to be empirical in nature and design. Second, these studies were required to use embodied physical action (EPA) robots. Third, studies were required to include measures of human or perceived robot personality. Fourth, studies must have included some interaction between at least one human and a robot to be eligible.

**FIGURE 1:**
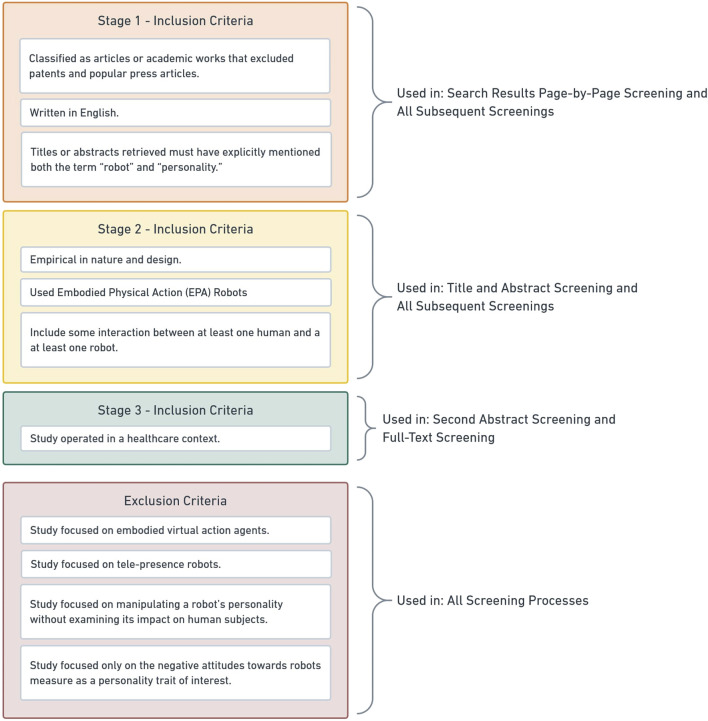
A summary of our screening criteria presented in a visual and format.

The final criterion used for including studies in this review at the tertiary level, required not only that the study meet all of the aforementioned eligibility requirements but also that the study operated within a health care context. For the purposes of this review, a health care context was any environment where the activities and interactions performed were directly related to an individual’s physical well-being. Studies were excluded if they 1) focused on embodied virtual action (EVA) (i.e. virtual agents), 2) focused on tele-presence robots, 3) focused only on manipulating perceived robot personality without examining its impact on a human, or 4) focused only on negative attitudes toward robots (NARS) as the personality trait of interest. The exclusion of studies that used the NARS scale was based on this scale’s use as a control variable in many studies (You and Robert, 2018a; Robert et al., 2020). A visual representation of our inclusion and exclusion criteria and where they were applied is visible in Figure 1.

### 3.5 Screening Procedure

Title screening was conducted manually in the revtools environment on the 1,069 unique entries previously identified. Screening was done only on the article title with author names and publication names hidden. Title screening was conducted based on the initial eligibility criterion. This screening identified 197 eligible studies.

Abstract screening was conducted manually in the revtools environment on the previously screened 197 studies. Abstracts were extracted from Google Scholar and manually added to the data-set utilized by revtools. This approach was adopted because Google Scholar has no native export system and the exporting of abstracts on behalf of “publish or perish” is incomplete and contains missing data. This screening utilized all previous eligibility criteria in addition to the secondary eligibility criteria. After identifying 84 studies that met our secondary eligibility criteria, we conducted abstract screening a second time using all previous eligibility criteria in addition to the final eligibility criteria. After this second abstract screening, we selected 13 studies for full-text screening.

In addition, we identified 50 other potential references from previously published review papers on the topic (Robert et al., 2020). All papers identified via this means were reviewed in the same way as the papers identified by our search (title and abstract screening) and with identical criteria. Ultimately, seven of the additional 50 references were found to be eligible for full-text screening.

**FIGURE 2:**
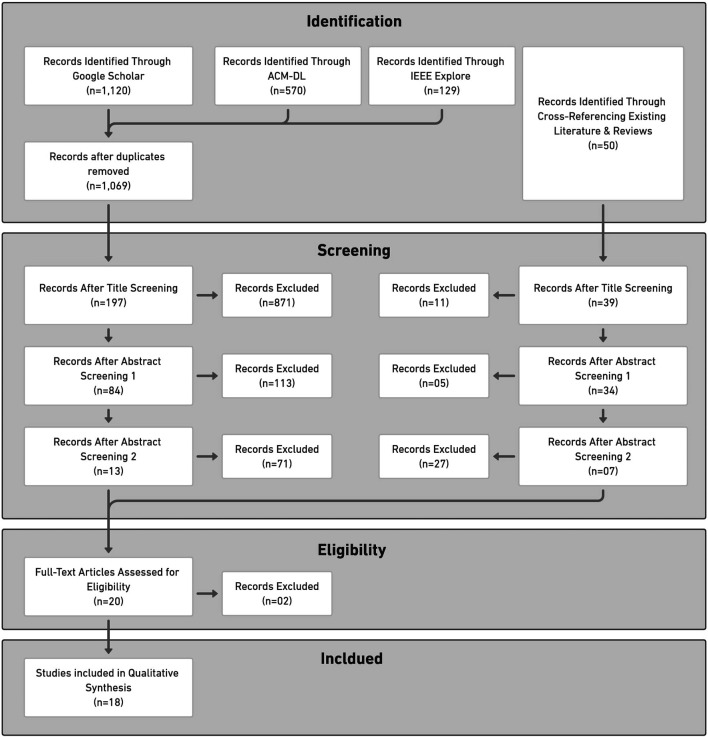
Prisma flow diagram of literature review process.

Full-text screening involved reading each of the 20 selected papers in detail to determine their suitability based on all previously listed criteria. After completing this screening, we excluded two more papers because they reported on the same study (Tapus and Matarić, 2008). The two excluded studies were Tapus et al. (2006) and Tapus et al. (2008). Figure 2 visually represents this review process and the associated counts.

## 4 Review Results

### 4.1 Publication Outlets

**FIGURE 3:**
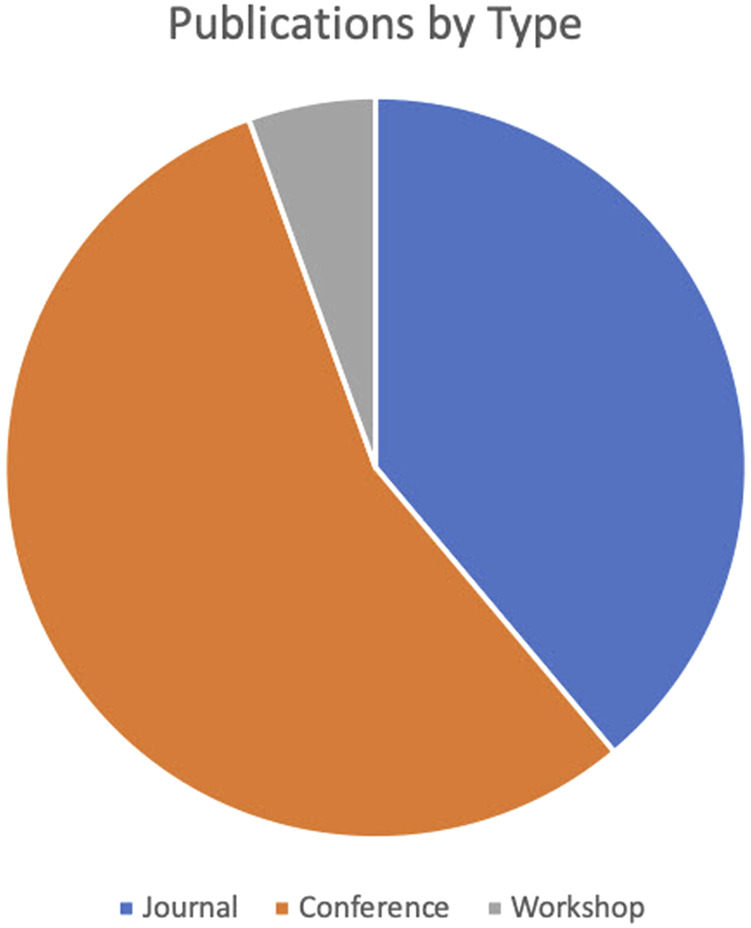
Publications by type.

The literature review search identified 18 total published papers met the criteria. These publications were primarily in conferences (10) and journals (7), with only one study appearing as a workshop paper. A breakdown of publications by type is presented in Figure 3. In terms of specific venues, there was no dominant single publication venue, with 8 of 19 studies being published in unique venues. However, four papers were published at the ACM SIGCHI conference and two were published in the International Journal of Human–Computer Studies.

**FIGURE 4:**
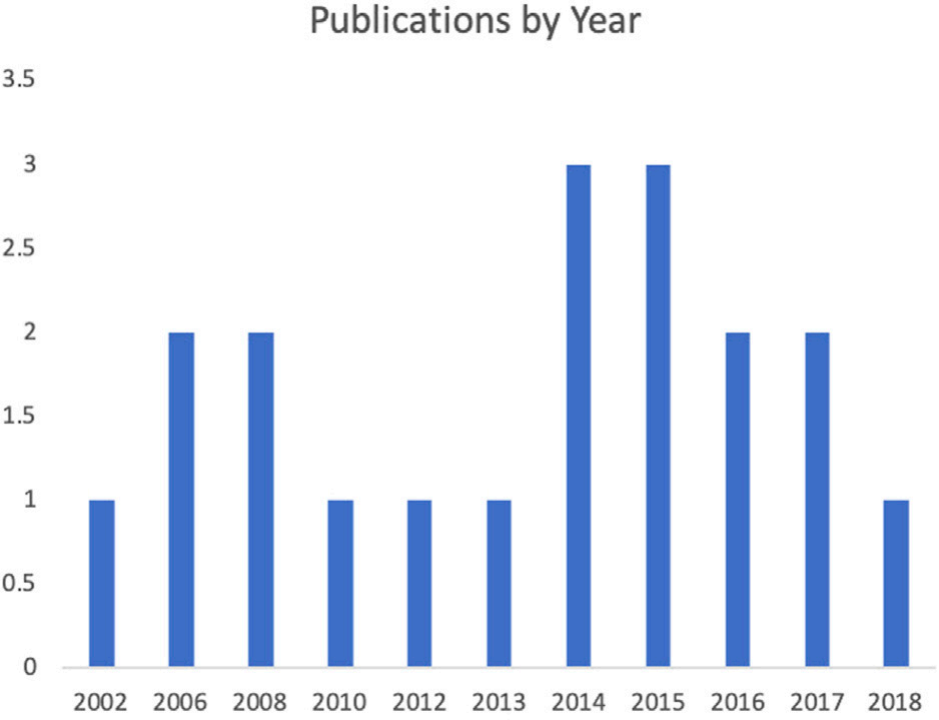
Publications by year.

Most publications were in outlets focused on human–computer interaction (5), human–robot interaction (3), interactive systems (2), and human factors, robotics, and controls engineering (2). The remaining studies ranged significantly, with two published in outlets focused on broad psychological subject matter, two published in outlets focused on aging and assistive technology, one published in an interdisciplinary open-source journal and the remaining paper published in an outlet focused on emotion, social signals, sentiment, communication. Notably, there was a lack of papers published in medicine-specific outlets. In terms of publication year, most studies were published between 2014 and 2018 as opposed to between 2002 and 2014. A breakdown of publications by year is in Figure 4.

### 4.2 Samples

#### 4.2.1 Participant Ages

**FIGURE 5:**
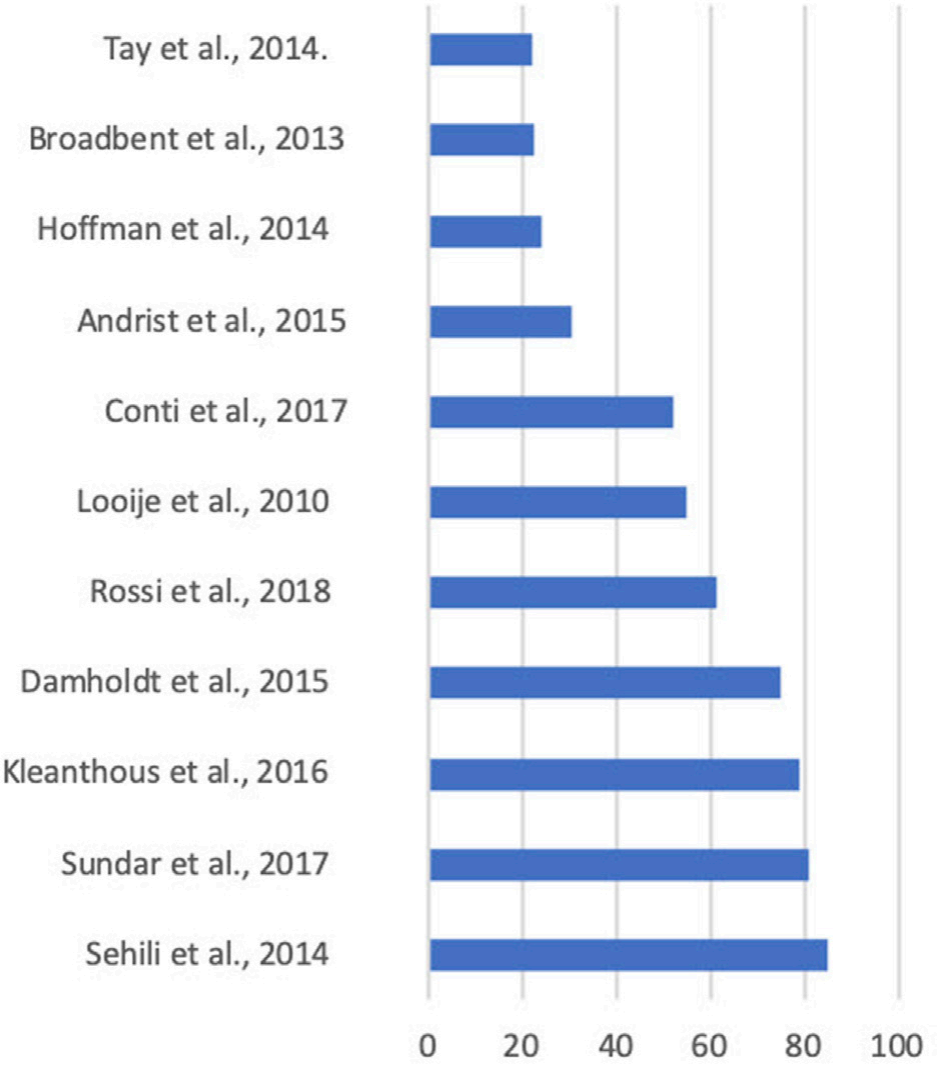
Average participant age by study.

The mean age across all studies was 47 and the standard deviation was 25. Specifically, six studies’ average ages fell between 18 and 44 years, three fell between 45 and 64 years, and four had subjects 65 years or older. This is a fairly representative age range across studies, as is evident in Figure 5. Notably, the large number of 65+ represented in this review is the result of a handful of studies taking place in rehabilitation or retirement communities. Given the location of this sample, it is possible that the 65+ population is not encompassing of the independently living 65+ populations. Notably, four studies in this review did not report age groups (Goetz and Kiesler, 2002; Powers and Kiesler, 2006; Tapus and Matarić, 2008; Weiss et al., 2012) and three reported only age ranges (Gockley and MatariĆ, 2006; Dang and Tapus, 2015; Cruz-Maya and Tapus, 2016).

#### 4.2.2 Gender

**FIGURE 6:**
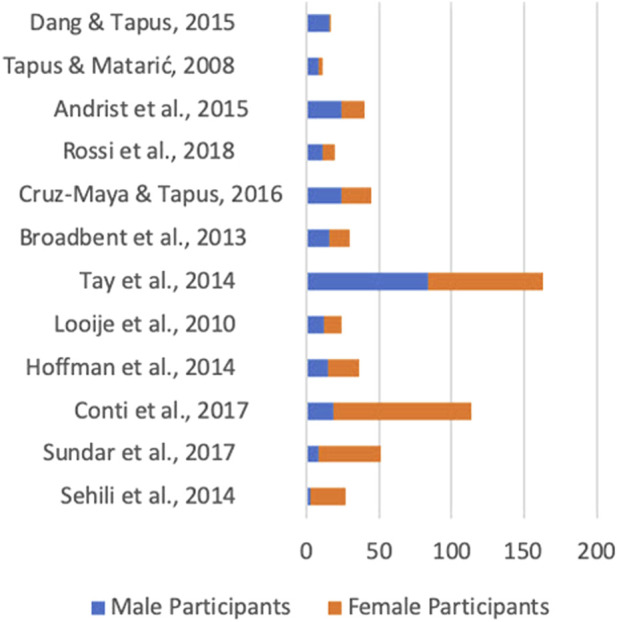
Gender balance by study.

Across all studies sampled, the percentage of women represented in this review is 57% and the percentage of men is 43%. Of all the studies, three did not provide gender information (Goetz and Kiesler, 2002; Powers and Kiesler, 2006; Damholdt et al., 2015) while one only stated the majority (Kleanthous et al., 2016). Notably, there was significant variation among studies, with some having more than 70% male samples and others having more than 70% female samples. This creates a scenario where the average distribution of men to women seems fairly balanced overall but in individual studies this distribution was uneven. Figure 6 demonstrates this trend, with the top half of studies represented having more men and the bottom having more women in their samples. There is evidence, as a result, that within this literature gender is not represented evenly in most studies and thus results of these studies are less generalizable than they might at first seem.

#### 4.2.3 Nation of Origin

Overall, samples were collected across all but one of the world’s major regions. Europe was the most frequently sampled region by far, with 64% (11) of reported samples. The remaining regions represented were the Middle East and Africa (2:12%), Asia (2; 12%), and North America (2; 12%). No samples were found to be from South America or Central America. Notably, the majority (10; 55%) of studies failed to provide region or country information in relation to their samples. In sum, three studies took place in more than one country while the remainder took place in the same country.

### 4.3 Level of Analysis

All studies were reviewed to determine their level of analysis. Generally, level of analysis for a particular study is at an individual, group, or organizational level. All studies included in this review were executed at the individual level, leaving the group and organizational dynamics not investigated.

### 4.4 Personality Traits

Overall, the literature examining personality in H-HRI employed five types of personality scales, which are shown in Table 4. However, the Big Five personality scale was commonly used either in whole or in part by the majority (8) of the reviewed studies. Alternative scales varied significantly among the remaining non-Big-Five studies, with two using scales either from or based on Wiggins and Broughton (1991) two using the NEO-EFI personality inventory, and one using the Eysenck Personality Inventory (EPI). The remaining studies used different scales from one another, and notably one study (Kleanthous et al., 2016) failed to provide details on the scale employed. Across all studies the most common dimension of personality studied was introversion vs. extroversion, which was measured in 13 of 18 studies.

**TABLE 4:** Personality traits and scales.

	Study	Scale	Personality traits					
			Extroversion	Agreeableness	Conscientiousness	Emotional stability	Openness	Other
Human Personality	Conti et al. (2017)	Big Five Questionnaire Caprara et al. (2007)	x	x	x	x	x	—
	Looije et al. (2010)	Big Five Questionnaire Van Vliet (2001)	x	x	x	x	x	—
	Sehili et al. (2014)	Big Five Traits Gosling et al. (2003)	x	x	x	x	x	—
	Cruz-Maya and Tapus (2016)	Big Five Goldberg (1990)	x	x	x	x	x	—
	Dang and Tapus (2015)	Big Five Goldberg (1990)	x	—	—	—	—	—
	Gockley and MatariĆ (2006)	Big Five Goldberg (1990)	x	—	—	—	—	—
	Damholdt et al. (2015)	NEO-EFI Costa and McCrae (1992)	x	x	x	x	x	—
	Andrist et al. (2015)	Big Five John and Srivastava (1999)	—	—	—	—	—	—
	Tapus and Matarić (2008)	EPI Eysenck and Eysenck (1968)	—	—	—	—	—	—
	Rossi et al. (2018)	NEO-PI-3 McCrae et al. (2005)	x	x	x	x	x	—
Perceived Robot Personality	Goetz and Kiesler (2002)	Big-Five John et al. (1991)	x	x	x	x	x	—
	Looije et al. (2010)	Other:New Measure	—	—	—	—	—	x
	Broadbent et al. (2013)	Other:Asch’s checklist of characteristics (Asch, 1961)	—	—	—	—	—	x
	Hoffman et al. (2014)	Other:Unnamed measure based on other works	—	—	—	—	—	x
	Powers and Kiesler (2006)	Other:Bem Sex Role Inventory Bem (1974)	—	—	—	—	—	x
	Sundar et al. (2017)	Other:New Measure	—	—	—	—	—	x
	Tay et al. (2014)	Wiggins Wiggins (1979)	x	—	—	—	—	—
	Weiss et al. (2012)	Wiggins Wiggins (1979)	x	—	—	—	—	—
	Andrist et al. (2015)	Big Five John and Srivastava. (1999)	x	—	—	—	—	—
	Tapus and Matarić (2008)	EPI Eysenck and Eysenck (1968)	x	—	—	—	—	—
	Kleanthous et al. (2016)	Not Provided	x	—	—	—	—	x

### 4.5 Outcomes

Outcome measures varied significantly and studies often employed more than one. In all there were 25 outcomes. The 25 outcomes can be grouped into four broad categories: performance, acceptance, social/emotional, and perceived robot personality. Performance accounted for 36% of the outcomes investigated. Generally, performance measures ranged from perceptual measures of how well the human or robot accomplished a task to objective measures represented by task scores or task time.

The second broad category of outcomes was acceptance, specifically acceptance of robots as health care providers. This category was made up of studies that looked at acceptance, usage time, preference, trust, distance, and satisfaction. Acceptance accounted for 36% of outcomes. The third category was that of social/emotional outcomes. This category encompassed studies of attachment, cooperativeness, empathy, friendliness, warmth, social presence, and likeability. This category accounted for 21% of outcomes. The fourth category was perceived robot personality, or perceptions of the robot itself. This category included measures of the human’s perceptions of the robot’s personality or degree of anthropomorphism. This outcome made up 7%. Table 5 provides an overview of studies and the outcomes they investigated.

**TABLE 5:** Outcomes.

Study	Outcomes			
	Performance	Social/Emotional	Acceptance	Perceived
		Outcomes		Robot personality
Andrist et al. (2015)	x	—	—	—
Broadbent et al. (2013)	—	—	x	x
Conti et al. (2017)	—	x	x	—
Cruz-Maya and Tapus (2016)	x	—	—	—
Damholdt et al. (2015)	—	x	—	—
Dang and Tapus (2015)	x	—	x	—
Gockley and MatariĆ (2006)	—	—	x	—
Goetz and Kiesler (2002)	x	x	—	—
Hoffman et al. (2014)	x	x	—	—
Powers and Kiesler (2006)	x	—	—	—
Kleanthous et al. (2016)	—	—	x	—
Looije et al. (2010)	—	x	x	—
Rossi et al. (2018)	x	—	—	—
Sehili et al. (2014)	—	—	x	x
Sundar et al. (2017)	x	—	x	—
Tapus and Matarić (2008)	—	—	x	—
Tay et al. (2014)	x	x	x	—
Weiss et al. (2012)	x	—	x	—

## 5 Themes

We identified three thematic uses of personality in the H-HRI literature: human personality, perceived robot personality, and human and perceived robot personality.

### 5.1 Theme 1: Participant (i.e. Patient) Personality

Studies that investigated human personality exclusively represented up to 39% of the studies. These studies investigated how participants’ personality impacted their interaction experience with the robot. Studies of this kind typically used participant personality as the independent variable and measured this personality via the Big Five personality traits. For example, Conti et al. (2017) measured human personality characteristics to determine whether different scores across the Big Five personality traits led to differences in the quality of interactions with the robot and ultimately the acceptance of the robot.

### 5.2 Theme 2: Health Care Robot (i.e. Provider) Personality

Studies that exclusively investigated robot health care personality represented up to 44% of the studies. These studies investigated how the robot’s personality impacted the quality of the patient’s interactions or experiences with the robot. For example, Goetz and Kiesler (2002) investigated how a robot’s perceived personality manipulated by its interaction style (playful vs. serious) impacted the human’s compliance with the robot and perceptions of the robot’s intelligence.

### 5.3 Theme 3: Participant (i.e. Patient) and Robot (i.e. Provider) Personality

Studies that investigated both human and perceived robot personality represented 17% of studies. These studies typically investigated how both the participant’s and the robot’s personalities impacted the quality of human-robot interaction. These studies varied significantly in their aims and approaches but overall focused on the impact of matching or mismatching the human and perceived robot personality on the quality of their interactions. For example, Looije et al. (2010) investigated the relationship between matching or mismatching a human’s personality traits with a robot’s perceived personality traits. In particular, they examined how matching/mismatching impacted a subject’s preferences for either an extroverted or introverted robot. Similar studies of this kind used various scales but focused on the measurement of extroversion and introversion.

## 6 Findings

Across the literature, results indicated a significant overall relationship between personality and human’s experiences with health care robots. Outcomes examined across studies were fairly evenly distributed among performance (36% of studies), acceptance (36% of studies), and social/emotional outcomes (21% of studies), but perceptions of a robot’s perceived personality or anthropomorphism were examined infrequently (7% of studies).

### 6.1 Theme 1: Participant (i.e. Patient) Personality

Studies investigating a patient’s personality and its relationship to HRI focused primarily on performance (3 studies) and acceptance (4 studies) outcomes. Additionally, they included social/emotional outcomes (2 studies), and patient’s perceptions of the robot outcomes (1 study). Table 6 summarizes these findings which we detail below.

**TABLE 6:** Patients personality traits impacts on outcomes by traits, where *↓* indicates a negative relationship and *↑* indicates a positive relationship.

Patient personality and performance outcomes		
Sig traits	Non-sig traits	Mixed
Agreeableness *↑* Rossi et al. (2018)	Extroversion Dang and Tapus (2015), Rossi et al. (2018)	Neuroticism Cruz-Maya and Tapus (2016), Rossi et al. (2018)
Conscientiousness *↑* Rossi et al. (2018)	—	—
Openness *↑* Rossi et al. (2018)	—	—

Patient Personality and Acceptance Outcomes		
Sig Traits	Non-Sig Traits	Mixed
Openness *↑* Conti et al. (2017)	Agreeableness Conti et al. (2017)	Extroversion Gockley and MatariĆ (2006), Dang and Tapus (2015), Conti et al. (2017)
—	Conscientiousness Conti et al. (2017)	Neuroticism Sehili et al. (2014) Conti et al. (2017)

Patient Personality and Social/Emotional Outcomes		
Sig Traits	Non-Sig Traits	Mixed
Extroversion *↑* Damholdt et al. (2015), Conti et al. (2017)	Agreeableness Damholdt et al. (2015), Conti et al. (2017)	Openness Damholdt et al. (2015), Conti et al. (2017)
Neuroticism *↓* Damholdt et al. (2015), Conti et al. (2017)	Conscientiousness Damholdt et al. (2015), Conti et al. (2017)	—

Patient Personality and Perceptions of Robot's Anthropomorphism		
Sig Traits	Non-Sig Traits	Mixed
Neuroticism *↓* Sehili et al. (2014)	—	—

#### 6.1.1 Performance Outcomes

Overall studies linking patient personality and H-HRI performance have found mixed results. In particular, Rossi et al. (2018) found that subjects high in agreeableness, conscientiousness, and openness to experience performed significantly better on a health-care-robot-assisted evaluation than those low in these traits. Extroversion, however, was found by Rossi et al. to be non-significant, which aligns with Dang and Tapus (2015). Specifically, Dang and Tapus found that performance on a robot-assisted task was not significantly different between extroverted and introverted participants.

Notably, studies on neuroticism found mixed results as Cruz-Maya and Tapus (2016) found neuroticism had a significant and positive relationship with performance while Rossi et al. (2018) found that it had no relationship with performance. Cruz-Maya and Tapus (2016) examined the performance of patients on a nutrition information test after receiving instruction from a robot. Results showed that male participants with higher neuroticism scored significantly lower than males with low neuroticism. Rossi et al. (2018), on the other hand, saw no significant difference in performance on their health care robot assisted psychometric evaluation between participants high versus low in neuroticism. One explanation for these conflicting results, however, may be the fact that Cruz-Maya and Tapus (2016) only reported a significant relationship between neuroticism and performance for male participants. This may imply that the same relationship may not be present for female participants or may not have an overall effect across the entire sample. The results of females or the overall sample were, however, not elaborated on in Cruz-Maya and Tapus (2016) making it difficult to draw firm conclusions about females or the entire sample. Future research may wish to re-examine the role of neuroticism and in doing so take into account gender as a potential interaction effect.

#### 6.1.2 Acceptance Outcomes

Several studies examined the impacts of human personality on acceptance of health care robots. Overall the relationship between human personality and H-HRI acceptance has been mixed. Across these studies, only openness to experience was found to be significantly related to acceptance. Specifically, Conti et al. (2017) found openness to experience as positively correlated with acceptance. Agreeableness and conscientiousness were also examined by Conti et al. (2017) but were found to be non-significant. The remaining traits of extroversion and neuroticism produced mixed results. Specifically, two authors found extroversion to be non-significant (Gockley and MatariĆ, 2006; Dang and Tapus, 2015) while one found extroversion had a significant and positive impact on the acceptance of robots (Conti et al., 2017). Similarly, neuroticism also produced mixed results with Conti et al. (2017) finding neuroticism as negatively impacting acceptance and Sehili et al. (2014) finding a non-significant effect. One possible explanation for these contradictory results may be related to sample sizes as Conti et al. (2017) utilized sizably more subjects than Dang and Tapus (2015), Gockley and MatariĆ (2006), and Sehili et al. (2014). Specifically, Conti et al. (2017) surveyed 114 subjects while Dang and Tapus (2015) surveyed 17 subjects, Gockley and MatariĆ (2006) surveyed 11 subjects, and Sehili et al. (2014) surveyed 27 subjects. It is therefore possible that significant effects only emerged when sample sizes were large enough to capture smaller effect sizes.

#### 6.1.3 Social/Emotional Outcomes

Several studies examined the impacts of human personality on social/emotional outcomes. Overall the relationship between human personality and social/emotional outcomes was mixed. Specifically, human personality was significantly related to social/emotional outcomes when it came to the traits of extroversion and neuroticism but not agreeableness or conscientiousness. Furthermore, openness to experience led to conflicting results. Of the traits that were found significant, extroversion was investigated by Conti et al. (2017) who examined the personality traits of teachers and their impact on the perceived social presence of a robot. Results showed a significant and positive correlation between extroversion and social presence where the more extraverted an individual was, the more likely that person appeared to attribute social presence to a robot. Consistent with these results, Damholdt et al. (2015) examined extroversion’s potential relationship with psychological relatedness and intimate-personal relatedness and found a positive correlation between subjects’ extroversion and these social/emotional outcomes.

Neuroticism (also referred to as emotional stability) was also significant but lead to a negative impact on social/emotional outcomes. For example, Conti et al. (2017) examined the impact of neuroticism and found a correlation between neuroticism and anxiety associated with robots, while Damholdt et al. (2015) found that higher neuroticism was negatively correlated with mental relatedness. Together these studies indicated a negative relationship between neuroticism and different social/emotional outcomes. In addition, Conti et al. and Damholdt et al. also found non-significant results related to agreeableness and conscientiousness’s impact on social/emotional outcomes. In short, both Conti et al. and Damholdt et al. found that neither of these traits was influential.

Two studies found contradictory results in relation to openness to experience (Damholdt et al., 2015; Conti et al., 2017). Specifically, Conti et al. (2017) found openness to experience to positively correlate with perceptions of robot sociability and beliefs that robots can be socially supportive. Damholdt et al. (2015), on the other hand, saw no significant relationships between openness to experience and social/emotional outcomes. A possible explanation for these contradictory results might be that though openness to experience could lead to seeing robots as sociable, it might not directly impact the degree to which an individual relates to the robots. Future work is still needed to examine these contradictions and determine to what degree human personality impacts other kinds of social/emotional outcomes.

#### 6.1.4 Patients’ Perceptions of the Robot’s Anthropomorphism

The effect of a human’s personality and their perceptions of robots was only examined by one author, namely, Sehili et al. (2014), who focused on neuroticism/emotional stability and its impact on anthropomorphic perceptions of robots. They found that humans high in neuroticism were more likely to anthropomorphize robots while those who were low in neuroticism were less likely to do so.

### 6.2 Theme 2: Perceived Robot Personality

Studies investigating perceived robot personality typically used measures of personalty separate from the Big Five personality traits. These studies focused on performance (7) followed by acceptance (7). Additionally, these studies investigated social/emotional outcomes (3). Notably, only one study investigated robots’ perceived personality as an outcome. Given the range of personality traits examined, results varied sizably. Table 7 summarizes these findings which we detail in the following sections of this paper.

**TABLE 7:** Robots’ perceived personality traits impacts on outcomes by traits, where *↓* indicates a negative relationship and *↑* indicates a positive relationship.

Perceived robot personality and performance outcomes			
Sig traits	Non-sig traits	Mixed	Sig unreported
Playfulness *↑* Goetz and Kiesler (2002), Sundar et al. (2017)	Responsiveness Hoffman et al. (2014)	—	—
Femininity *↑* Tay et al. (2014)	—	—	—
Extroversion *↑* Weiss et al. (2012), Tay et al. (2014)	—	—	—

Perceived Robot Personality and Acceptance Outcomes			
Sig Traits	Non-Sig Traits	Mixed	Sig Unreported
Playfulness *↓* Sundar et al. (2017)	—	—	Friendliness *↑* Kleanthous et al. (2016)
Femininity *↑* Tay et al. (2014)	—	—	Directness *↑* Kleanthous et al. (2016)
Extroversion *↑* Weiss et al. (2012)	—	—	—

Perceived Robot Personality and Social/Emotional Outcomes			
Sig Traits	Non-Sig Traits	Mixed	Sig Unreported
Playful *↑* Goetz and Kiesler (2002)	—	—	—
Responsive *↑* Hoffman et al. (2014)	—	—	—
Feminine *↑* Tay et al. (2014)	—	—	—

Perceived Robot Personality and Perceptions of robot’s personality			
Sig Traits	Non-Sig Traits	Mixed	Sig Unreported
Sociability *↑* Broadbent et al. (2013)	—	—	—
Amiability *↑* Broadbent et al. (2013)	—	—	—

#### 6.2.1 Performance Outcomes

Across studies examining performance, a handful of perceived robot personality traits were shown to be significant. Overall, seven studies looked at robots personality traits and examined how these impact performance outcomes. In particular, extroversion, and femininity were found to have significant and positive impacts on performance (Weiss et al., 2012; Tay et al., 2014). Playfulness, on the other hand, was examined by Goetz and Kiesler (2002) and Sundar et al. (2017) both of whom found that had a significant and negative relationship with performance. Specifically, robots with serious personalities produced a higher performance score than robots with playful personalities. Sundar et al., explained these findings by highlighting the importance of considering a robot’s role. They found that if the robot was assigned to an assistant role, the more playful it was, the better performance measures were, whereas a robot in a companion role who was playful led to lower performance outcomes. Only one study examined responsiveness. This was Hoffman et al. (2014) who found that more or less responsive robots had no bearing on individual’s perception of the robot’s performance.

#### 6.2.2 Acceptance Outcomes

Studies linking robot’s personalities to robot acceptance each looked at unique personality traits with no studies examining the same two traits. In particular, studies examined playfulness, femininity, friendliness, directness, and extroversion. Of these characteristics, playfulness, femininity, and extroversion were found to be significant and to have positive associations with acceptance outcomes (Weiss et al., 2012; Tay et al., 2014; Sundar et al., 2017). Notably, Kleanthous et al. (2016) failed to report significance tests values but claimed to have observed a positive association between friendliness and acceptance outcomes, and a negative association between directness and acceptance outcomes.

#### 6.2.3 Social/Emotional Outcomes

Studies examining social/emotional outcomes each focused on different personality traits but generally found a significant association across these traits. Specifically, Goetz and Kiesler (2002) found that participants who interacted with a playful robot were happier than those that interacted with a serious robot. Additionally, Hoffman et al. (2014) found that a more responsive personality produced significantly higher social perceptions. Finally, Tay et al. (2014) found that more feminine-seeming robots produced more positive responses. Ultimately, it appears that even though no studies examined social/emotional outcomes for the same personality traits, personality is linked to social/emotional outcomes.

#### 6.2.4 Patients’ Perceptions of the Robot’s Perceived Personality

Only one author examined the relationship between perceived robot personality traits and patients’ perceptions of robots. In this case, Broadbent et al. (2013) considered perceptions of a robot’s anthropomorphism/human-likeness as the independent variable and their assignment of personality traits as the dependent variable/outcome. The authors found that the more anthropomorphic the robot, the more that individuals assigned the robot positive personality traits (Broadbent et al., 2013). Positive personality traits in this case were the sociability and amiability of the robot. In short, results showed a significant and positive relationship between anthropomorphism and these perceived personality traits.

### 6.3 Theme 3: Patient and Perceived Robot Personality

Three studies in this review looked at both patient’s and robots’ personalities. Andrist et al. (2015) examined personality’s relationship with performance, while Looije et al. (2010) and Tapus and Matarić (2008) investigated personality’s impact on acceptance. In addition, Looije et al. examined human and robot personalities interactions with social/emotional outcomes. Table 8 summarizes these findings.

**TABLE 8:** Robot and patient personality traits impacts on outcomes.

Patient and perceived robot personality traits impacts on performance outcomes			
Sig Traits		Effect	Study
Robot	Patient	—	—
Introverted	Introverted	Sig Positive	Andrist et al. (2015)
Extroverted	Extroverted	N.S	Andrist et al. (2015)

Patient and Perceived Robot Personality Traits Impacts on Acceptance Outcomes			
Sig Traits		Effect	Study
Robot	Patient	—	—
Extroverted	Extroverted	Sig Positive	Tapus and Matarić, (2008)
Introverted	Conscientious	Sig Positive	Looije et al. (2010)

Patient and Perceived Robot Personality Traits Impacts on Social/Emotional Outcomes			
Sig Traits		Effect	Study
Robot	Patient	—	—
Extroverted	Conscientious	Sig Negative	Looije et al. (2010)

#### 6.3.1 Performance Outcomes

Only one study examined performance outcomes as they relate to matching or mismatching personality traits in H-HRI. In particular, Andrist et al. (2015) compared performance ratings for robots that were either extroverted or introverted and examined any differences in ratings on the basis of a subject’s degree of extroversion. Results of this study found that the degree to which a robot’s perceived personality matched a patient’s (g.g., introverted robot to introverted subject) was influential but only among introverts. Specifically, introverted humans working with an introverted robot accomplished a robot assisted puzzle faster than introverts partnered with an extroverted robot. The same relationship was, however, not found between extroverted robots and extroverted humans (Andrist et al., 2015).

#### 6.3.2 Acceptance Outcomes

Two studies examined the effects of patient and robot personalities on acceptance. First, Looije et al. (2010) examined the impact of a more social (extroverted) vs. a less social (introverted) robot and how this impacted patients with varying degrees of conscientiousness. They found that highly conscientious patients had higher degrees of acceptance of less social (introverted) robots than highly social (extroverted) robots. The second study, Tapus and Matarić (2008) examined the effect of matching or different personalities between patients and robots and how these matches or mismatches impacted acceptance. Findings from this study indicated that extroverts tended to have higher acceptance for extroverted robots and that introverts had higher acceptance for introverted robots.

#### 6.3.3 Social/Emotional Outcomes

For social/emotional outcomes, only one study investigated the effects of both patient and perceived robot personality in combination. Namely, Looije et al. (2010) examined the perceived degree of a robot’s sociability (highly sociable robots vs. non sociable) and the degree of patient conscientiousness (high or low). Given sociability’s relationship with extroversion vs. introversion, highly sociable robots can be considered extroverted while non-sociable robots can be considered introverted. Results indicated a relationship where the more conscientious a participant was and the more sociable (extroverted) the robot was, the less likely the patient liked the robot after interacting with it.

### 6.4 Summary of Findings

The findings of this paper can be organized into three overarching insights with regards to promoting beneficial outcomes such as: performance, acceptance and social/emotional reactions. It should be noted that there is also empirical evidence with regard to other findings, but these insights represent the most consistent and generalizable results across the literature.

Findings 1: Generally, patient personality traits such as agreeableness and consciousness were positively associated with beneficial outcomes (Rossi et al., 2018). However, the relationship between patient personality traits such as openness, neuroticism and extroversion and beneficial outcomes were mixed. More specifically, sometimes they were positively associated with beneficial outcomes (Damholdt et al., 2015; Conti et al., 2017; Rossi et al., 2018) while at other times they were negatively associated with such outcomes (Gockley and MatariĆ, 2006; Sehili et al., 2014; Damholdt et al., 2015; Dang and Tapus, 2015; Cruz-Maya and Tapus, 2016; Conti et al., 2017).

Finding 2: Robot personality traits such as feminine, extroverted, responsive, sociable and amiability were positively associated with beneficial outcomes (Weiss et al., 2012; Broadbent et al., 2013; Hoffman et al., 2014; Tay et al., 2014). The impact of playfulness as a robot personality was mixed. At times, when a robot had a playful personality it was positively related to beneficial outcomes (Goetz and Kiesler, 2002; Sundar et al., 2017) while at other times it was negatively related (Sundar et al., 2017). It should be noted friendliness and directness were not reported.

Finding 3: Matching robot and patient personality based on extroversion or introversion were positively associated with beneficial outcomes. However, miss-matching patient and robot personalities had mixed effects. More specifically, miss-matching was sometimes positive and sometimes negative (Looije et al., 2010).

## 7 Comparison and Contrasting of Findings With Health Care Literature

Personality appears to be influential in both the human–human health care and the H-HRI domains. Although these two domains represent distinct research foci, several overlapping sub areas allow us to make comparisons and contrasts between the two. To do so, we examine theme 1 (patient personality) and theme 2 (provider personality) findings and their similarities and differences. In particular, findings on patients’ personalities in human–human health care and H-HRI related to performance are consistent at certain times and inconsistent at others. Notably, results related to acceptance are largely inconsistent with each other, while results related to provider’s personality and performance—human or robot—appear to have limited overlap. In the latter case, however, where overlap is present, the findings appear to be consistent. Table 9 provides a summary of these findings which we discuss in detail below.

**TABLE 9:** Comparison of human-human health care studies and H-HRI studies on personality.

Patients’ personality traits and health care performance outcomes			
Personality Trait	Health Care HRI	H-H Health Care	Comparison
Agreeableness	Sig Positive	Sig Positive	Consistent
Conscientiousness	Sig Positive	Sig Positive	Consistent
Extroversion	N.S.	Sig Positive	Inconsistent
Neuroticism	Mixed	Sig Negative	Inconsistent
Openness	Sig Positive	—	—

Patient’s Personality Traits and Acceptance Outcomes			
Personality Trait	Health Care HRI	H-H Health Care	Comparison
Agreeableness	N.S.	—	—
Conscientiousness	N.S.	—	—
Extroversion	Mixed	—	—
Neuroticism	Mixed	—	—
Openness	Sig Positive	Sig Negative	Inconsistent
Social Desirability	—	Sig Positive	—
Detachment	—	Sig Positive	—
Optimism	—	Sig Positive	—
Stress Susceptibility	—	Sig Negative	—
Pessimism	—	Sig Negative	—

Provider Personality and Performance Outcomes			
Personality Trait	Health Care HRI	H-H Health Care	Comparison
Agreeableness (BFI)	—	Sig Positive	—
Conscientiousness (BFI)	—	Sig Positive	—
Extroversion (BFI)	Sig Positive	Sig Positive	Consistent
Neuroticism (BFI)	—	Sig Negative	—
Openness (BFI)	—	Sig Positive	—
Playfulness	Sig Negative	—	—
Femininity	Sig Positive	—	—
Responsiveness	Non-Sig	—	—

### 7.1 Patient Personalities and Performance

We examined performance outcomes across studies in both the human–human health care and H-HRI domains. In the context of H-HRI, patient performance comprised how well human participants performed on assessments or retained health-related information after it was presented by a robot. In the human–human health care context, performance largely related to a patient’s responses to treatment and/or the patient’s post-treatment quality of life (QoL). Comparing patients’ personality traits in the human–human health care and H-HRI literature, it appears that both agreeableness and conscientiousness have significant and positive effects on performance outcomes, whereas mixed results emerge between extroversion and neuroticism.

One possible explanation for the inconsistent results between the human-human health care and H-HRI domains in terms of extroversion and performance might revolve around the sample sizes utilized. In particular, the sample sizes of studies in the H-HRI domain were 21 participants in the case of Rossi et al. (2018) and 17 in Dang and Tapus (2015). These are relatively small samples, especially when compared to the samples in studies in the human-human health care domain, which numbered 151 in Kim et al. (2013) and 66 in Koh et al. (2014). It is therefore possible that increases in sample sizes might produce results consistent with those found in the human-human health care domain.

Beyond extroversion, studies in the human-human health care and H-HRI domains also produced inconsistent results related to performance and patients’ neuroticism. Specifically, within the H-HRI domain Cruz-Maya and Tapus (2016) found results similar to studies in the human-human health care domain while Rossi et al. (2018) found inconsistent (non-significant) results. One possible explanation for the inconsistency in Rossi et al. might again revolve around sample size because this study only utilized a sample of 17 participants, whereas studies in the human-human health care domain utilized samples between 66 and 802 participants (Kim et al., 2013; Koh et al., 2014; Victorson et al., 2016). Additionally, the population in Rossi et al. (2018) was primarily elderly adults which might also account for these inconsistent results because studies in the human-human health care domain recruited more diverse samples.

### 7.2 Patient Personalities and Acceptance

Along with performance outcomes, we examined acceptance-related outcomes across both the human–human health care and the H-HRI domains. For studies in the H-HRI domain, acceptance outcomes were related to usage time, preferences, trust, distance, and satisfaction, whereas studies in the human–human health care domain focused primarily on satisfaction. Notably, studies in the human–human health care domain largely focused on different personality traits from those examined by studies in the H-HRI domain. As a result, a lack of overlap between these domains emerged. Openness to experience bridged this gap, with both domains showing results related to this personality trait; however, openness to experience produced inconsistent results between the two domains.

One explanation for openness to experience’s inconsistent results might be attributable to gender differences. Specifically, within the human–human health care domain there appears to be a focus on male samples, whereas within the H-HRI domain there appears to be a focus on female samples. For example, in the human–human health care domain, subjects were examined after they underwent treatment for prostate cancer, which primarily affects male populations (Mohler et al., 2010). Given the lack of specification as to the gender of the participants, one can confidently assume the sample was predominantly male. In the H-HRI domain, however, Conti et al. (2017) noted that their sample was 84.2% female.Therefore, it is possible that openness to experience is influenced by gender to some degree. Ultimately, more examination is needed before firm conclusions can be made.

### 7.3 Provider Personalities and Performance

Provider performance has been examined across both the human–human health care and the H-HRI research domains. Specifically, within the human–human health care domain there is a focus on the Big Five personality traits of agreeableness, conscientiousness, extroversion, neuroticism, and openness to experience, whereas the H-HRI domain focuses on a range of outcomes and alternative sets of personality traits. Notably, one commonality between these research domains is the importance of extroversion.

Extroversion generally showed a significant and positive relationship with performance outcomes. Specifically, when human health care providers possessed high degrees of extroversion their performance was rated as higher, and when robotic providers were perceived as having high degrees of extroversion, the robots’ performance was rated similarly high. This commonality might relate to some aspects of health care work that favor extroverted workers (human or otherwise) over introverted workers. For example, a study examining extroversion and workplace performance found that task significance (impact of work on lives of others) and task variety (degree to which job requires multiple tasks) are significant moderators between extroversion and job performance (Dietl and Kombeiz, 2021). This study utilized human health care workers as one of its samples and found significant and positive moderating effects for both task significance and task variability but noted that, uniquely in the health care context, when task significance was lower, extroverts actually performed worse (Dietl and Kombeiz, 2021).

## 8 Discussion and Opportunities

Despite the importance of personality in the H-HRI literature, there are several major gaps. Next, we present research opportunities (ROs) in the literature based on important gaps. These include research opportunities related to study samples, national biases, group-level analysis, and human and robot personalities. We focused on these issues because they represent several of the most salient yet addressable issues going forward.

### 8.1 RO 1: Sample

We identified three primary issues related to the sample across the studies in the review: sample size, sampling participants ages 65+, and the wide disparity with regard to gender diversity.

#### 8.1.1 Size

The vast majority of studies included fewer than 50 participants in their sample, with three studies standing apart (Powers and Kiesler, 2006; Tay et al., 2014; Conti et al., 2017) in having out-size samples. The mean sample size excluding these large-sample studies averaged only 23.8, making generalization of results rather limited because such small samples are prone to sampling error (Blaikie, 2004). In that the majority of studies (83%) identified in this review had relatively small participant counts, there is an opportunity for new studies to provide additional strength to these existing findings by including additional participants and increasing their relative sample size.

#### 8.1.2 65+ Participants in Diverse Settings

There is a need to examine the impacts of personality in H-HRI with participants older than 65 in settings other than assisted-living/medical-residency programs. Many individuals older than 65 live home alone and might have different challenges from those living in assisted-living/medical-residency programs. Therefore, there is a need to both identify those challenges and explore the role of personality in H-HRI. This is an unexplored area of study in personality in H-HRI.

#### 8.1.3 Gender

Across the studies the issue of gender imbalance was much more problematic than it might appear, with 57% women vs. 43% men in total. However, nearly two-thirds of the studies reviewed had wider gender imbalances. This makes it difficult to generalize their findings across both populations. Additional studies are needed with properly balanced samples ensuring equal representation of men and women. In doing so, these studies would provide insights that are more generalizable across populations.

### 8.2 RO 2: National Biases

Europe was the most frequently sampled region by far, with 64% of reported samples. The remaining regions represented were the Middle East and Northern Africa (12%), Asia (12%), and North America (12%). No samples were found from South America, Central America, or sub-Saharan Africa. Notably, the majority (55%) of studies failed to provide region or country information in relation to their samples. However, if we used the location of the authors of the papers, the breakdown appears similar, with North America (28%) increasing in size and Europe (52%) as well as the Middle East and Northern Africa (8%) decreasing in size. Asia (12%) remained consistent. Once again, we still find a lack of studies with populations from South America, Central America, or sub-Saharan Africa. That being said, we should acknowledge that our focus on English-language-only articles could in part explain the lack of studies in South America, Central America, or sub-Saharan Africa. To partly address this shortcoming, we conducted a post hoc informal review for non-English-language papers on this topic. Unfortunately, we failed to identify any additional studies. Therefore, there appears to be a gap in studies with samples from South America, Central America, and sub-Saharan Africa, or at least in English-language publications.

### 8.3 RO 3: Level of Analysis

No studies focused on personality in health care HRI investigated group-level interactions. Humans and robots in a health care context are certain to have one-on-one interactions, but these are not the only kind of interactions. For example, health care services are normally carried about by a team or group of health care workers rather than one individual. Therefore, a group-level analysis might assist in the investigation of teams and teaming between humans and robots (You and Robert, 2018a; b, 2019, 2017). The lack of investigation beyond the individual level of analysis provides an opportunity for researchers.

### 8.4 RO 4: Human and Perceived Robot Personality

At present, two studies investigated the interplay between humans’ and robots’ personalities in H-HRI. This stream of research is particularly important for two reasons. One, in reality both the human’s and the robot’s personalities have to be taken into consideration. Therefore, understanding the interplay between them is likely to provide important insights that can be generalized into valuable design recommendations. Two, there is a growing debate in the HRI community on whether it is better to match human and perceived robot personality or mismatch them to achieve better interactions (Robert, 2018; You and Robert Jr, 2018; Zhang et al., 2019; Robert et al., 2020). Answering this question in the context of H-HRI would be valuable. A limitation, however, is a lack of studies focused on robot’s personalities when compared to studies focused on human personalities. Therefore, it is important that more researchers examine robot’s personalities to make comparisons more robust.

## 9 Conclusion

Robots are becoming an important way to deliver health care across the world, and personality is vital to understanding their effectiveness. To establish what we know and identify what we do not know in this area, we conducted a review involving 1,069 articles. This review identified 18 studies that met the eligibility criteria. Specifically, we examined studies that provided the results of empirical research focused on human personality and interactions with embodied physical action robots in a health care context. We organized the results of this investigation into three overarching themes and highlighted the gaps within these themes. This paper is an important starting point in establishing an understanding of personality in H-HRI. Future research is needed to build on this review and expand our understanding of personality in H-HRI. Specifically, another review is needed to determine whether there are any differences in the role of personality for human interactions with EPA robots versus human interactions with virtual agents/telepresence robots in health care. In addition, future work should also consider perceptions of robots beyond acceptance as well as how these findings may change across different domains beyond healthcare.

## Data Availability

The original contributions presented in the study are included in the article/Supplementary Material, further inquiries can be directed to the corresponding author.
