# Supplementary material for: A Systematic Review of Human and Robot Personality in Health Care Human-Robot Interaction
Source: Front Robot AI. 2021 Sep 17;8:748246. doi: 10.3389/frobt.2021.748246 (PMC8484868; doi:10.3389/frobt.2021.748246)
Supplement: Supplementary file 1 [file DataSheet1.PDF]

Supplementary Material

1 SUPPLEMENTARY TABLES

1.1 Funding by Study

Study	Robot Utilized	Type
Andrist et al. (2015)	Meka robot	Humanoid
Broadbent et al. (2013)	Peoplebot healthcare robot	Machine-Like
Conti et al. (2017)	Video of NAO	Humanoid
Cruz-Maya and Tapus (2016)	Kompai Robot	Humanoid
Damholdt et al. (2015)	The Telenoid	Humanoid
Dang and Tapus (2015)	Video of NAO	Humanoid
Gockley and MatariĆ (2006)	Pioneer 2-DX	Machine-Like
Goetz and Kiesler (2002)	Nursebot	Humanoid
Hoffman et al. (2014)	Travis	Machine-Like
Kleanthous et al. (2016)	GrowMeUp System	Machine-Like
Looije et al. (2010)	icat	Humanoid
Powers and Kiesler (2006)	Unspecified Robot	Humanoid
Rossi et al. (2018)	Pepper	Humanoid
Sehili et al. (2014)	NAO	Humanoid
Sundar et al. (2017)	Home Mate Robot	Machine-Like
Tapus and Matarić (2008)	Pioneer 2-DX	Machine-Like
Tay et al. (2014)	Unspecified Robot	Humanoid
Weiss et al. (2012)	Pictures of NAO	Humanoid

Table S1. Table summarizing the specific robots utilized across studies.

1.2 Robots Utilized by Study

Study	Funded By
Andrist et al. (2015)	Chateaubriand Research Fellowship.
Broadbent et al. (2013)	This research was funded by a University of Auckland Research Excellence award.
Conti et al. (2017)	European Union's H2020 research and innovation program under the MSCA-Individual Fellowship grant agreement no. 703489.
Cruz-Maya and Tapus (2016)	CONACYT-French Government n.382035.<br>EU Horizon2020 ENRICHME project grant agreement no. 643691C.
Damholdt et al. (2015)	VELUX FOUNDATION<br>Department of Health and Assisted Living Technologies (Municipalityof Aarhus),
Dang and Tapus (2015)	French National Research Agency (ANR) via the Chaire D'Excellence program 2009 (Human–Robot Interaction for Assistive Applications).
Gockley and MatariĆ (2006)	NSF Graduate Research Fellowsh<br>NSF Grants #IIS-032914 and #IIS-0121426.
Goetz and Kiesler (2002)	NSF Grants # 0085796 and #1120180
Hoffman et al. (2014)	FP7 Marie Curie CIG #293733, and<br>Binational Science Foundation Grant #2011381.
Kleanthous et al. (2016)	European Commission within the H2020-PHC-2014, (Grant Agreement: 643647)
Looije et al. (2010)	IOP-MMI Senter Novem via the Dutch Ministry of Economics
Powers and Kiesler (2006)	NSF Grant #IIS-0121426
Rossi et al. (2018)	European Union's H2020, MSCA-IF no.703489.<br>MIUR's PRIN2015 "UPA4SAR" project n. 2015KB-L78T.
Sehili et al. (2014)	Not Specified
Sundar et al. (2017)	KORUS Tech Program (KT-2010-SW-AP-FSO-0004) as part of the International Collaborative R&D Program of the Korea Ministry of Science, ICT and Planning (MSIP).
Tapus and Matarić (2008)	USC Women in Science and Engineering (WiSE) Program and the OkawaFoundation
Tay et al. (2014)	Not Specified
Weiss et al. (2012)	Not Specified

Table S2. Table summarizing funding sources by study where provided
